# Dyspepsia

**Published:** 1881-01

**Authors:** 


HALL'S

### JOURNAL OF HEALTH

AND

MISCELLANY.

E. H. GIBBS, A. M., M. D. - - EDITOR.

Founded in 1854, and published Monthly without Interruption since that Time.

### DYSPEPSIA.

WHEN we consider that this complaint may be induced by causes diametrically opposite, it is not surprising that very few entirely escape its unpleasant symptoms.

Whatever interferes with the regular processes of digestion causes dyspepsia. It may be so slight as to escape the notice of the patient; or, if he experiences some discomfort in the region of the stomach, more or less headache, and perhaps occasional palpitation of the heart, he is apt to attribute it to other causes, and to continue a course of diet and habits of life that brought on the trouble, until the symptoms become too aggravated for further endurance, and then he consults a physician. There is no organ of the body so liable to disorder as the stomach; no organ that is more abused. The general health of body and mind depends on its regular and healthy action, and, in order to act regularly and healthfully, the food supply must be of the most wholesome kind, and in quantity just sufficient to supply the waste caused by those processes we call life.

If mind and body are habitually active, the waste is greater and a larger supply of food is essential; hence, persons whose business requires regular and moderate exercise out of doors, and who are temperate in all things, seldom suffer from dyspepsia, and are usually cheerful and hopeful. Over-eating is, however, less frequently a cause of aggravated dyspepsia than insufficient food, crude and indigestible and innutritious food, rum or tobacco. The reason is obvious. The gourmand eats too much because his food is palatable and nutritious and his appetite stronger than his will. The stomach digests and makes good use of all that is required, and thus the waste is supplied, and the system is nourished and strengthened. The individual is too indolent to create a demand for the excess he has eaten by proper exercise, and this excess ferments and irritates the stomach, and causes the generation of gases, and the patient suffers from flatulence and heart-burn. Reasonable self-restraint afterward will cause these symptoms to disappear, and a cautious person will not often repeat such imprudences. But it is the great army of miserable, improvident, ignorant and dissipated persons who suffer most severely. Their food, when wholesome, is often insufficient, but usually it is of the crudest sort, badly cooked, and swallowed before it is half masticated. The stomach finds little that is fit to supply the demands of the system, and the bulk of it acts as an irritant to that organ, and to the entire intestinal canal. This irritation alone further debilitates a system that is weak, and stimulants are often resorted to, at first perhaps as temporary expedients to relieve the suffering of body and mind. These soon lose their effect, but the habit, unfortunately, is generally continued to the destruction of its victim.

Medicine alone will not cure dyspepsia. Whatever means or agencies tend to improve the general health are the best remedies, not only for dyspepsia but for all other diseased conditions.

Even in health it would be well to observe the following rules :

*First.*—Strict personal cleanliness.

*Second.*—Regular daily out-door exercise, and regular and sufficient movement of the bowels once a day.

*Third.*—From seven to nine hours sleep every night in a perfectly ventilated room.

*Fourth.*—Just enough wholesome and nutritious food, nicely prepared, to fully supply the requirements of the system ; this to be followed at dinner by fresh ripe fruit.

*Fifth.*—Rich pastry, puddings and pies, should be avoided, but plain rice, tapioca, sago, bread or Indian puddings may generally be eaten by dyspeptics.

*Sixth.*—Avoid spirits and tobacco. The habitual use of either of these poisons will inevitably bring on dyspepsia, in the train with other evils.

*Seventh.*—Eat regularly three times a day, thoroughly masticating every particle of food.

*Eighth.*—Keep constantly occupied in some useful and profitable business, leaving no time to brood over real or imaginary troubles. "Cross no bridges till you come to them."

The usual cause of dyspepsia is error in eating.

The principle of cure is comprehended in the above rules.

As a temporary relief for some of the annoying symptoms of dyspepsia, many things have been recommended, and it may be well to mention a few of them. Calamus, or sweet-flag, often affords immediate relief. Half a teaspoonful of table salt in six tablespoonfuls of water half an hour before meals. A teaspoonful of large white mustard seed to be eaten every morning. A glass of milk or butter-milk. Plain soda water, or half a teaspoonful of bicarbonate of soda in half a tumbler of water. An orange eaten after each meal will sometimes prevent attacks of dyspepsia.

Ordinary dyspepsia can not long exist if the bowels are regular; and their condition demands the promptest attention. It will generally be found that there is a torpid condition of the liver, with more or less constipation.

So long as this condition exists recovery is impossible, because the blood is being constantly contaminated by the poisonous matters that are too long retained in the system. There are two plans of treatment: One is by regulating the diet in such manner as to give nature an opportunity to relieve itself of the burden; the other is to aid nature with certain remedies. The latter plan is the shortest, and, as a rule, whatever accomplishes any object soonest is the best; therefore relieve the liver by taking two to four "liver pills" at bed time, and afterward the bowels may be kept regular by using a suppository every night at bed time, or in severe cases a suppository morning and night. This treatment is safe, and in a majority of cases effectual, provided always that the foregoing rules are properly observed.

---

As a test for organic impurities in water, the use of a dilute solution of tannic acid has recently been suggested—the test solution to contain five per cent. of tannin, and five parts of it to be added to one hundred of the water. If organic matters be present, a pellicle or scum will rapidly form; this scum formation can be recognized by the immediate appearance of an iridescence or play of colors, and the growth of fungus vegetation can be detected without a microscope, by the little bubbles of carbonic acid which collect around the edges of the surfaces. In every sample of water where this turbidity or scum is formed, or where a fungoid growth occurs soon after the addition of the tannin solution, organic matters are certainly present. When these organic matters have been destroyed by evaporating, heating, etc., no such turbidity or fungoid growth occurs on addition of the tannin solution.

